# Minimization of torque pulsations by using a novel fuzzy controller in SRM drives for EV applications

**DOI:** 10.1016/j.heliyon.2023.e14437

**Published:** 2023-03-15

**Authors:** S. Kudiyarasan, N. Sthalasayanam, Vijayalakshmi Karunakaran

**Affiliations:** a BHAVINI, DAE, Kalpakkam, Tamilnadu, 603127, India; b SRM Institute of Science and Technology, Chennai, Tamilnadu, 600089, India

**Keywords:** Electric vehicle, Fuzzy controller, Switched reluctance motor, Sliding mode control, Torque pulsations

## Abstract

In recent years, the applications of Changed Reluctance Motors have expanded, from control system stepping motors to high torque e-vehicle applications. High-speed operation and a light-weight driving motor are required for an effective electric vehicle design. Switched reluctance motor (SRM) is ideal for use in electric vehicles due to its low torque-to-weight ratio and magnet-free rotor design. The increased torque ripple is the most serious issue with switching reluctance motors. The optimization technique is used to optimize switching controllers in this study, and a comparison is made between a sliding mode controller (SMC) with a modified reaching law and a new fuzzy controller (FC). The magnitude of torque ripple is simulated and compared for both controllers using a MATLAB simulink model. The proposed innovative fuzzy controller model significantly improved torque performance and reduced torque ripples based on simulation results.

## 1 Introduction

BRUSHED and commutator-based machines have fallen out of favor due to the need for frequent brush replacement. Despite the fact that brush less direct current (BLDC) Motors offer simple control, the larger torque ripple during commutations [1] and inferior torque performance of PMSM are the main disadvantages when compared to BLDC Motors. Permanente magnet synchronous motor (PMSM) enables accurate torque and speed control. However, PMSM Motors have several significant drawbacks, including: Magnetic Deterioration of Permanent Magnets at High Temperatures and High Speeds, Motor construction requires rare-earth materials, which are scarce and have environmental consequences, and High Speed Operation is limited due to the presence of permanent magnets in the rotor, which has poor mechanical strength. Switched Reluctance Motors, on the other hand, are gaining popularity and are ideal for electric vehicle (EV) applications because they provide more benefits than PMSM and BLDC.

The winding and magnet-less rotor construction simplify the design while also eliminating the shortcomings of PMSM. It has a high torque-to-weight ratio and a low moment of inertia. All existing direct torque control and vector control algorithms demonstrated for Induction Motor control can be adapted to SRM for improved torque and speed performance. Due to the unipolar nature of stator current injected only during rising inductance interval for supplying necessary motoring torque results in discontinuous input current and thus high input THD and low power factor.

A midpoint converter topology based on dual output converters is proposed in the literature [2] for power factor correction during discontinuous inductor current operation, particularly at low speeds. The voltage following methodology used in the converter reduces the input line current THD. Another disadvantage of SRM drives is the high level of vibration and acoustic noise caused by changing radial forces between the stator and rotor during the commutation interval [3].

The SRM Drives are doubly salient drives, meaning they have saliency in both the stator and the rotor, resulting in non-linear magnetic flux couplings. The non-linear nature of SRM Drives becomes more apparent at higher speeds, making excitation/demagnetization control non-uniform and difficult across a wide speed range. As a result, torque ripples in SRM drives are extremely high. Machine design is one of three methods for reducing torque ripple in SRM drives currently available. The magnetic properties of the SRM have inherent nonlinearity. The control is complex, and the main challenge is to find the best and optimum switching angle for maximum torque performance while also focusing on reduction of torque ripple and negative torque generation [4]. The genetic algorithms are also gaining popularity as they can be employed to optimize the switching angles for effective torque performance [5]. A lower number of phases in SRM architecture results in larger torque ripple due to differences in aligned and unaligned inductances [6]. As a result, multiphase SRM or a high number of stator poles is preferred [7]. Proposes a magnetic equivalent circuit (MEC) technique for simulating the magnetic flux and torque performance of rotor segmented axial field SRMs. To understand flux linkage changes and propose a machine design solution for the torque pulsations issue in SRM, the MEC approach to machine modelling and design can simulate machine performance over a wide range of operating ranges and load torque circumstances. The second method is to employ novel power electronic converter topologies [8]. SRM performance is affected by converter topology as well as control methodology. For SRM control, the asymmetric bridge converter provides better performance and a simpler architecture [9]. The asymmetric half bridge converter topology is preferred over other converters such as the C-dump circuit and the N+1 Power electronic converter because it provides independent phase control and has lower torque pulsations. The third method is to use controller design, Torque Sharing Function (TSF), Direct Torque Management (DTM) and Direct Torque Control (DTC), for independent torque and speed control as well as superior dynamic torque performances [10].

Fuzzy controllers, sliding mode controllers, model adaptive predictive control, neural and genetic algorithm based controllers are used to reduce torque pulsations in SRM drives. To reduce torque ripple, the Direct Torque Control algorithm used in SRMs [11] uses higher current in non-effective rotor positions while simultaneously limiting maximum torque per ampere (MTPA). Improved direct instantaneous torque control methods, combined with torque sharing functions, produce better results; however, complex control is required [12]. Furthermore, the difficulty of controlling SRMs at low and high speeds necessitates two distinct techniques. At high speeds, late magnetization reduces torque, whereas late demagnetization results in a negative torque value. Based on the actual motor speed, supply voltage, and current constraints, the study suggests ideal start and stop times, as well as periods of excitation and de-excitation. Because traditional PI Controllers are ineffective at controlling nonlinear control systems, they are not recommended for SRM Drives [13]. Ref. [13] Proposes a switching variable proportional desaturation PI regulator, which eliminates integral saturation issues in PI regulators and provides improved dynamic performance and speed control stability at all stages of operation.

However, the inherent flaws of mathematical models must be investigated, as well as dynamic performance stability at higher operation speeds and torque ripple pulsations. Ref. [14] proposes a sliding mode controller (SMC) with a new reaching law to effectively reduce speed deviations in SRMs, as well as an anti-disturbance sliding mode observer (AD-SMO) for significant reductions in torque ripple, which is high especially at higher speeds due to high non-linearity in the Flux and rotation angle relationship. However, the proposed SMC and AD-SMO did not account for thermal management constraints in converter circuits, particularly at higher speeds, or the optimization required in control aspects. The application of a cost function rather than a hysteresis function to the selection of voltage vectors in the targeted sector of SRM operation during excitation and demagnetization periods for a reference torque, with the goal of optimizing the selection for required flux linkage. A simple PID controller is generally not used for SRM Control. Optimal tuning algorithms such as local Uni model sampling (LUS) and spotted Hyena optimizer (SHO) are being designed and the approach is best evaluated [15]. A novel hybrid controller can achieve best performance for any steady state error transient disturbance by combining traditional P control for steady state and fuzzy controller for transient state [16]. The most impartment quality of the controller should be minimizing the inherent chattering effect even during abnormal conditions [17]. In our concept, a near-real-time fuzzy model is developed, which demonstrates superior torque performance at both steady and transient states. Constant gain PI controllers are not suitable for high-performance control as SRM drives cause oscillations in different operating regions, which can be overcome by implementing SMC or a fuzzy controller [18].

## 2 Selection of SRM configuration and its power electronic converter topology

**Fig. 1:**
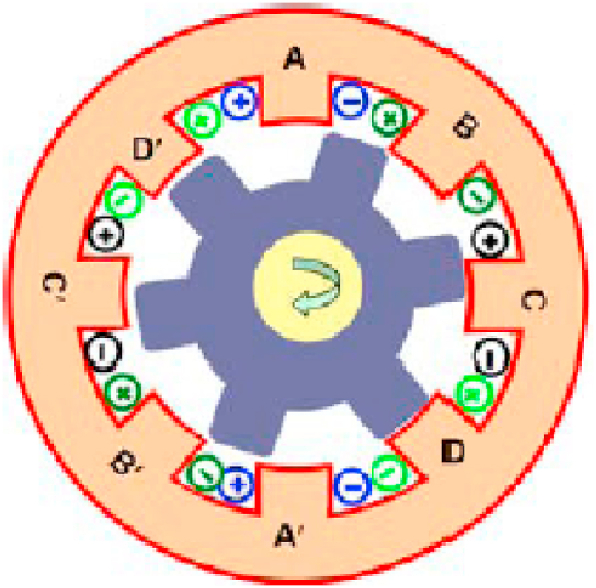
Constructions of 8/6 stator and rotor of SRM.

**Fig. 2:**
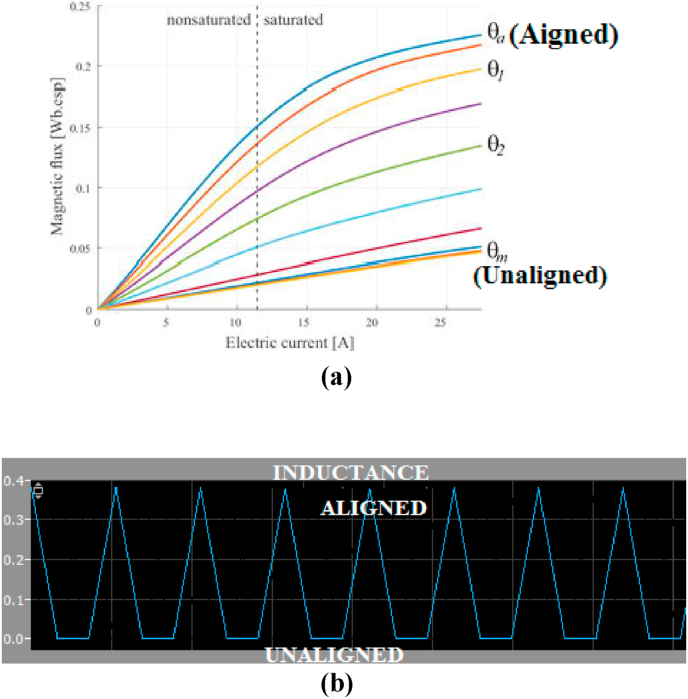
(a)Magnetic flux and current for various positions of SRM. (b)Inductance waveform showing aligned and unaligned variations.

SRM is founded on the concept of variable reluctance. The poles of the stator are wound, while the rotor has a laminated iron core with no winding. When the stator phase winding is turned on, magnetic field formed in the stator attracts the rotor iron, and the rotor orients itself to the stator in the minimum reluctance position. For modelling and simulation, an SRM with 8 stator poles and 6 rotor poles (8/6 SRM) as shown in Fig. 1 is used. Inductance variation (dL/dθ) occurs when the rotor rotates from an unaligned to an aligned position, as shown in Fig. 2a. Equation can be used to calculate the resultant torque (1) produced by SRM.

(1) $$T=\frac{1}{2}i^{2}\frac{dL}{d\theta }$$

The inductances change as the rotor rotates from its unaligned to its aligned positions as shown in Fig. 2b. Because current is squared, the direction of current has no effect on torque generation. When producing motoring torque, the fluctuation or slope of inductance should be positive. Thus, current is injected for phase excitation only during the positive inductance slope period.

Fig. 1 shows that each phase consists of four poles that are simultaneously excited by an external DC power source, which forces the pole from unaligned position θm to aligned position θa, the step angle ε can be arrived as per Eq. (2).

(2) $$\text{The}\;\;\text{step}\;\text{angle}\;\text{or}\;\text{Stroke}\;\varepsilon =\frac{2\pi }{\text{qNr}}=7.5^{\circ }$$

where, q is 8 No. of Phases and Nr is 6 No. of rotor poles.

**Fig. 3:**
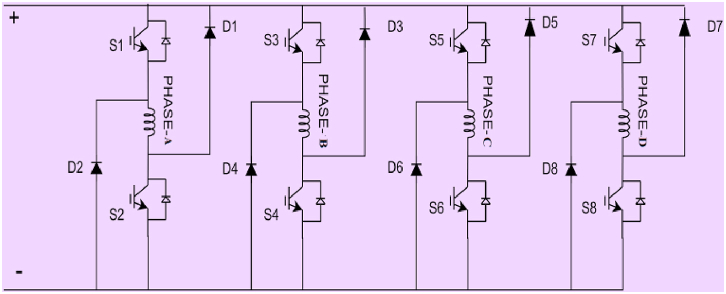
Asymmetric Half Bridge Converter circuit diagram.

The asymmetric half bridge converter shown in Fig. 3 is the most common converter topology for switched reluctance motor drive. Each phase circuit contains IGBT switches and two diodes. The 8/6 SRM converter topology consists of 8 IGBT switches and 8 Diodes.

**Fig. 4:**
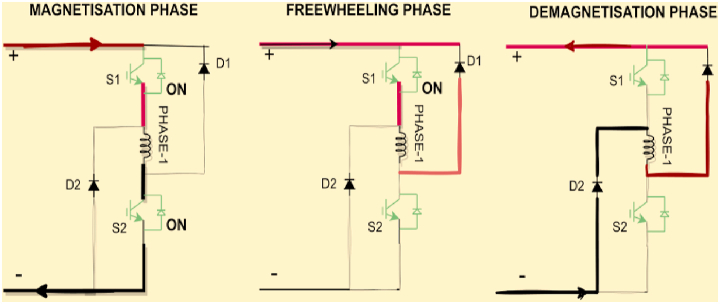
Operating modes of SRM with AHB Converter.

The asymmetric half bridge (AHB) converter has high fault tolerance and torque performance [19]. Both the S1 and S2 devices are turned on during motoring or magnetization mode, and a DC voltage is applied to the winding. When S2 is turned off, the winding current freewheels through S1 and D1, and demagnetization occurs through D1 and D2 when both IGBTs S1 and S2 are turned off. Fig. 4 depicts the modes graphically. The converter topology selected in this paper is asymmetric half bridge converter.

**Fig. 5:**
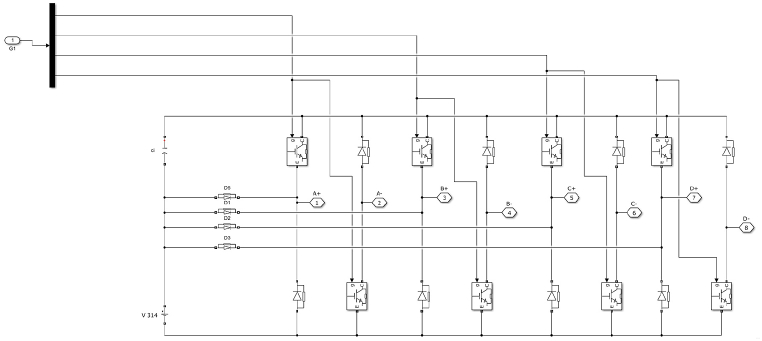
Power Electronic AHB Converter topology MATLAB Block.

The Converter topology selected in this paper is Asymmetric half bridge converter. The MATLAB Model is shown in Fig. 5.

### 2.1 Mathematic modeling of SRM

SRMs geometry and torque generation mechanism are unique, posing significant challenges in machine modelling. Because of the dual saliency in both the stator and the rotor, the air gap fluctuates greatly, implying that the machine flux linkage is a nonlinear function of rotor position. SRMs also operate in a magnetic saturation region. This complicates machine modelling and control.

The torque equation of a motor and load can be expressed as Eq. (3).

(3) $$\text{Te}\;\left(i,\theta \right)=\text{TL}+B\omega +J\frac{d\omega }{\text{dt}}$$

The Electrical torque of SRM as derived from the above Eq. (3),

(4) $$Te\left(i,\theta \right)=\frac{1}{2}i^{2}\frac{dL}{d\theta }$$

**Fig. 6:**
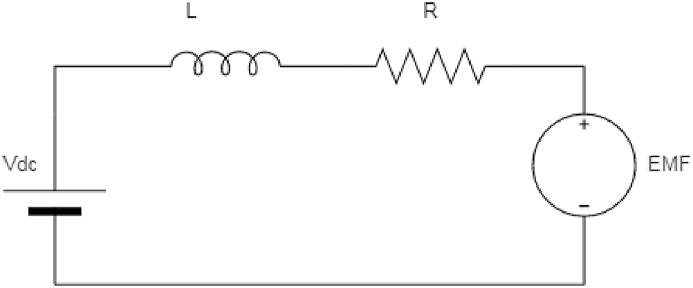
Simplified equivalent circuit.

Because SRM is non-linear as shown in Eq. (4), a simplified model is usually used. As a result, ignoring the mutual inductances between the phases results in a simplified equivalent circuit, as shown in Fig. 6.

The Voltage Equation can be given by Eq. (5),

(5) $$V=\text{iR}+L\left(i,\theta \right)\frac{\partial i}{\partial t}+i\frac{\partial L\left(i,\theta \right)}{d\theta }$$

(6) $$\text{The}\;\text{Rotational}\;\text{component}\;\text{is}\;\text{back}\;\text{KMF},e=i\left(\frac{\partial L\left(i,\theta \right)}{d\theta }\right)\omega$$

Back EMF is a linear function of rotational speed by using (5), (6). As the motor rotates, the EMF is induced in the phase windings. The resulting EMF opposes the applied excitation voltage to the phase windings, limiting the rate of change of phase current. As a result, the rotational back EMF is a crucial concept in understanding the phase current dynamics in SRM. Without taking into account the effects of resistance, the equation can be expressed as follows: As the EMF is a linear function of rotor speed, as expressed by equation. The rate of change of current is primarily determined by the motor’s speed from Eq. (7).

(7) $$\frac{\left(V-e\right)}{L}=\frac{di}{dt}$$

At low speeds, the induced voltage has no effect on the rate of change of current. As a result, the high current will result in significant copper losses in the windings, as well as thermal losses. Limit, the reference current chopping control is modified to ensure safe operation within thermal limits as well as torque production that meets the torque command.

**Fig. 7:**
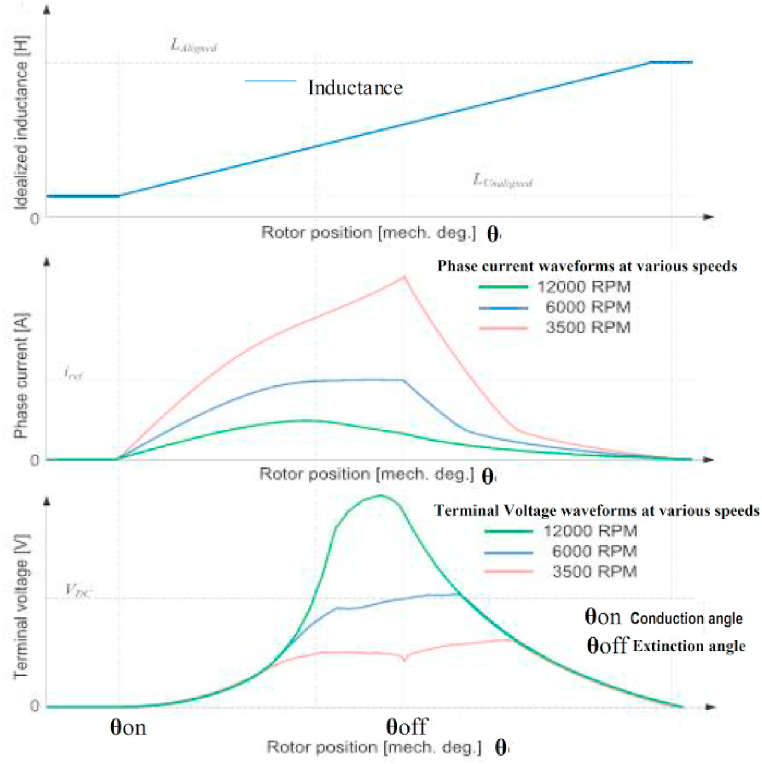
Voltage, Phase current and inductance waveforms of SRM as a function of rotor position.

**Fig. 8:**
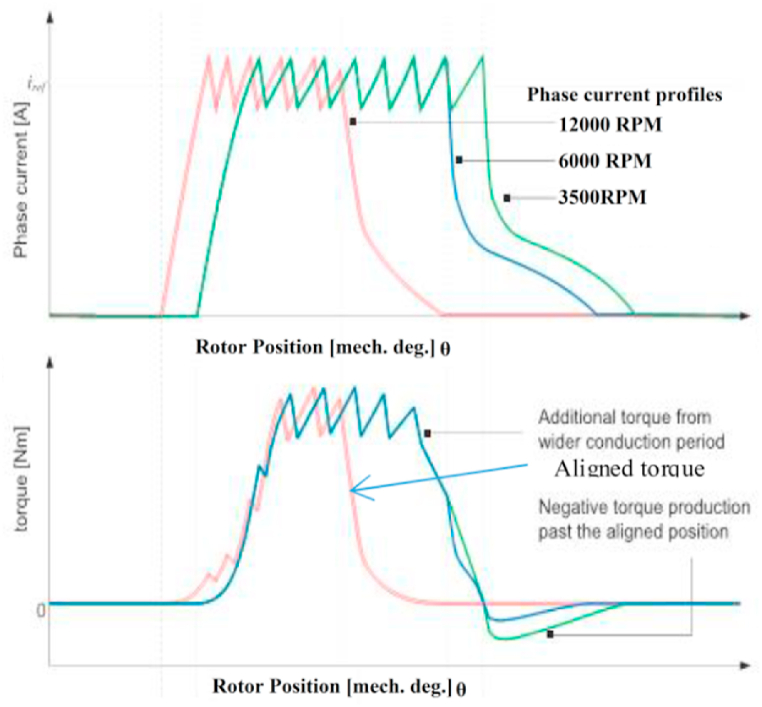
Torque and Phase current waveforms of SRM as a function of rotor position.

Fig. 7 depicts the inductance, phase current and terminal voltage as a function of rotor angular position. It can be observed that, even when a positive DC-link voltage is applied at higher speeds, the current decays automatically as the induced rotational EMF voltage exceeds the value of the DC-link voltage, causing phase current to decay. The difference between the Turn-on (**θ**on) and Turn-off (**θ**off) angles is the conduction angle. The optimal conduction angle must be chosen in order to achieve the desired torque performance and torque quality [20]. Torque is achieved at low speeds by controlling the current rise with PWM of the DC voltage; however, at higher speeds, the current rise is largely controlled by back EMF, and thus torque performance is largely dependent on the conduction angle. The greater the negative torque produced, the longer the conduction period as a result of the delayed turn off angle as shown in Fig. 8 depicting the phase current and torque waveforms as a function of rotor angular position.

The Torque to current conversion equation shall be expressed as given by Eq. (8),

(8) $$Te\left(i,\theta \right)\frac{1}{2}i^{2}\frac{dL}{d\theta }$$

$$i=\sqrt{\left(2\;\text{Tk}\left(i,\theta \right)\right)/\text{Lk}}\;\text{Where}\;\text{Lk}=\frac{\partial L\left(i,\theta \right)}{d\theta }$$

### 2.2 SRM control system

**Fig. 9:**
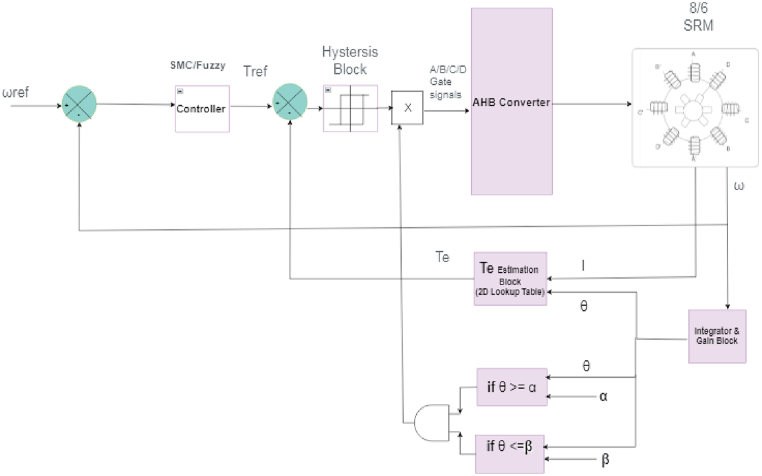
Block diagram of SRM Controller.

A switched reluctance motor’s control system is based on mathematical modelling. The outer speed control loop and the inner current controller loop are the two control loops. The encoder mounted in the switched reluctance motor provides the motor speed, and the speed encoder signal (velocity) is translated to rotor position by the discrete time integrator block. The speed controller compares the reference speed to the encoder’s speed feedback, and the error is processed by the controller block, which is a sliding mode controller or controller, and the controller’s output sets the torque reference. The Block diagram is shown in Fig. 9.

**Fig. 10:**
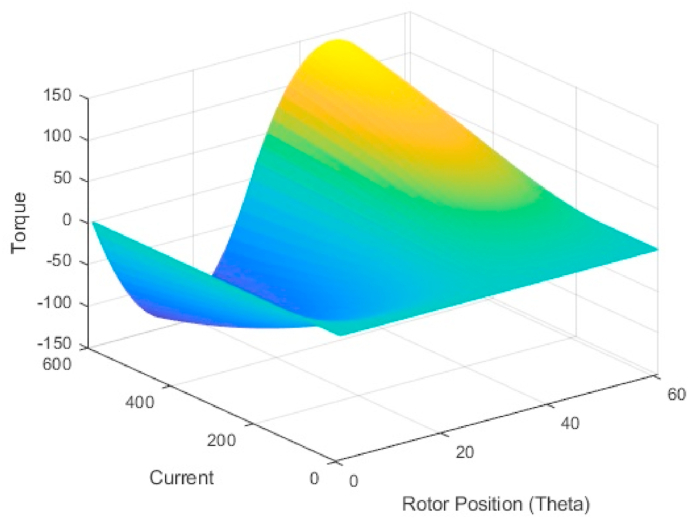
Estimated torque surface viewer w.r.t to current and Rotor position θ.

A two-dimensional lookup table Fig. 10 is used to calculate the actual torque, which expresses torque as a function of excitation current and rotor position. The estimated torque is compared to the controller’s reference torque, and the resulting current is processed by the current hysteresis block. The rotor’s position is compared to alpha (turn on angle) and beta (turn off angle).

Only when the rotor position is within the switching angle period are pulses sent to IGBT devices in the AHB Converter. The peak current at low speeds must be controlled to be at some commanded value i_ref_ in order to prevent the current from exceeding the thermal limits and to keep the machine running smoothly. A hysteresis band is represented by I_up_ and I_lo_ which can be expressed by Eqs. (9), (10). The tolerance of the hysteresis band can be expressed as μ.

(9) I_up_ = I_ref_ (1 + μ)

(10) I_lu_ = I_ref_ (1 − μ)

**Fig. 11:**
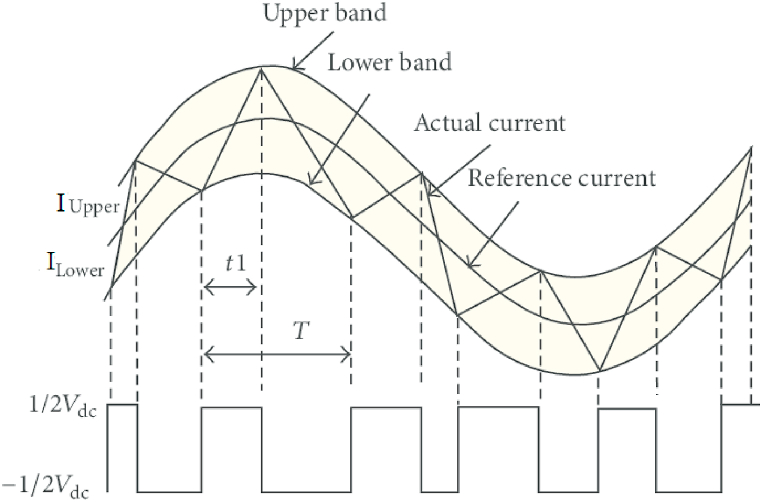
Hysteresis current controller for SRM

The phase inductance and the width of the hysteresis band at any given time determine the switching frequency of the hysteresis current controller as shown in Fig. 11. As a result, the control system at SRM operates at a variable switching frequency. Fundamentally, if the current’s rate of change is higher at a given instant, the current will diverge from the hysteresis band faster, causing the current controller to respond by switching the device faster. A slower rate of current change, on the other hand, indicates that switching will take longer.

## 3 Design of proposed sliding mode controller

Sliding mode control is one of the Robust Control strategies for achieving a stable equilibrium point in a control system. The equilibrium point is located on a sliding plane in SMC control. The system’s controlled trajectory is far from the plane, and regardless of its initial conditions, a series of control actions added to the system will bring it to the sliding plane or sliding surface. When the trajectory reaches the plane, it will glide along it until it reaches the equilibrium point O.

### 3.1 Governing mathematical equations

The Governing differential equations of a switched reluctance motor is given by Eqs. (11), (12),

(11) $$\left(\frac{d\theta }{d\tau }\right)=\omega$$

(12) $$\left(\frac{d\omega }{\text{dt}}\right)=\frac{1}{J}\left(\text{Te}-T_{L}-B\omega \right)$$

As the motor torque is the function of rotor position angle and current, Eq. (12) could be elaborated into (13),

(13) $$\left(\frac{d\omega }{\text{dt}}\right)=\frac{1}{J}\left(\sum_{j=1}\text{Tej}\left(\Theta j,\text{ij}\right)-T_{L}-B\omega \right)$$

where ω is angular speed, TL is load torque, B is viscous fraction coefficient and J is the inertia constant respectively.

$$\text{Considering},x1=\omega \;\text{and}\;x2=\frac{d\omega }{\text{dt}}$$

(14) $$x2=\frac{1}{J}\left(\sum_{j=1}\text{Tej}\left(\Theta j,\text{ij}\right)-T_{L}-\text{Bx}1\right)$$

The first derivative of x2 given in Eq. (14) can be represented by Eq. (15),

(15) $$\dot{x}2=\frac{1}{J}\left(\dot{T}e-\text{Bx}2\right)$$

(16) ex1 = x1 − x1d

where ex1is the speed error expressed as difference of measured (x1) and reference speed (x1d) as represented in Eq. (16).

The phase variable state equation can be simplified asper the (LTI) continuous-time system, ẋ = Ax + Bu as shown in Eq. (17).

(17) $$\left[\begin{array}{c} {ė}_{x1}\\ {ė}_{x2} \end{array}\right]=\left[\begin{array}{cc} 0 & -1\\ 0 & -a \end{array}\right]\left[\begin{array}{c} e_{x1}\\ e_{x2} \end{array}\right]+\left[\begin{array}{c} 0\\ b \end{array}\right]U$$

$$\text{where},a=\frac{B}{J};\;b=\frac{1}{J\tau }$$

U is the control output of SMC Controller. After the effect of integrator, it is the total reference torque of SRM.

### 3.2 Sliding surface

**Fig. 12:**
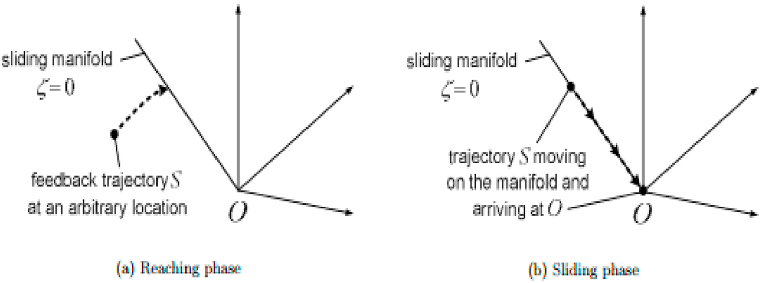
Sliding plane and reaching plane.

Now, let us assume that there exist sliding mode control law U which forces the phase trajectory to slide on the hyperplane S on a phase plane as shown in Fig. 12 for c > 0, λ∈R is given by Eq. (18),

(18) S(x, t) = (Ce_x1_ + e_x2_)

U is a control signal that, regardless of drive system parameter alterations, is utilized to govern the speed error dynamics. Eq. (19) is the mathematical description of the sliding line:

(19) $$S=c\left(\omega -\omega_{\text{ref}}\right)+\frac{d\left(\omega -\omega_{\text{ref}}\right)}{dt}$$

where *c* is a constant greater than 0. Sliding surface can be expressed in the form of error variable by Eq. (20).

(20) Ṡ = Cė_x1_ + ė_x2_

To maintain on a sliding surface, S = 0;

Ce_x1_ = −e_x2_

Ce_x1_ = −ė_x1_

ė_x1_ = −Ce_x1_

(21) e_x1_(t) = e^−ct^e_x1_(0) or ω = ω_ref_ (1 − e^−ct^)

Thus after reaching the sliding mode of system, Speed is only related with the parameters c as clearly arrived in Eq. (21). As long as c > 0, then the $\underset{t\to \infty }{\lim}\;\omega =$ ω_ref_, System is stable. If c values are higher, the response is faster.

In order to verify the stability of the reaching law, the Lyapunov function *V* = *s*^2^/2 is chosen.

The most important aspect of designing a sliding mode controller is to find a control strategy for the plant input U (t) such that,

$\frac{dV\left(s\right)}{dt}<0$, This condition should be regardless of the spatial position of the system state. To satisfy the existence condition of the sliding-mode speed controller, the following Eq. (22) must be satisfied:

(22) $$\underset{S\to 0}{\lim}\;s\frac{ds}{dt}<=0$$

The control legislation U (t) is intended to satisfy the preceding condition.When we use the control strategy, the system state is dragged towards the stable hyperplane. S(x, t) = (c e + ė).

**Fig. 13:**
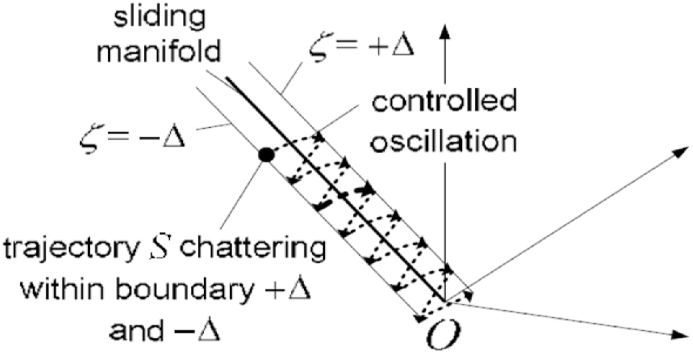
Hysteresis band in sliding manifold.

The system state will arrive at the hyperplane surface in a finite amount of time. When it reaches the surface, it slides along it towards the equilibrium point.

$$u\left(t\right)=\left\{ \begin{array}{c} U^{+}\;\;\;\;if\;S\left(x,t\right)>\Delta \\ U^{-}\;\;\;\;if\;S\left(x,t\right)<-\Delta \\ Previous\;state\;Otherwise \end{array}\right.$$

where Δ is a value arbitrarily small or the hysteresis band The addition of a hysteresis band with the boundary conditions *S* = Δ and *S* = *−*Δ, as shown in Fig. 13, provides a form of hysteresis control without control chattering.

**Fig. 14:**
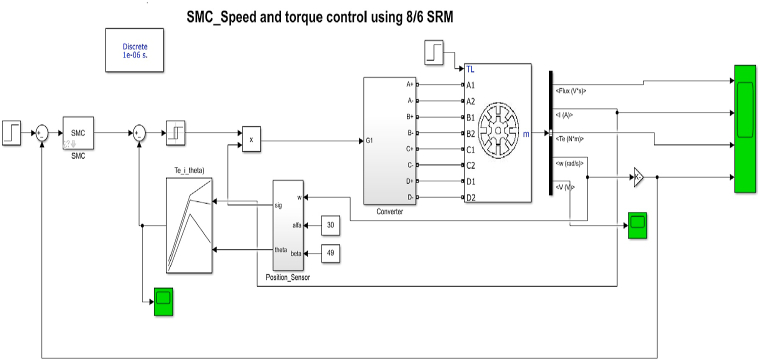
SRM control with siding mode controller simulink model.

The MATLAB Simulink model for SMC Control of 8/6 SRM drive is developed as shown in Fig. 14. The speed encoder feedback signal and current feedback is obtained for closed loop control. The speed is converted to Rotor position angle through position estimator block. The sliding mode controller is developed for the switching function (s) and control function U(t).

**Fig. 15:**
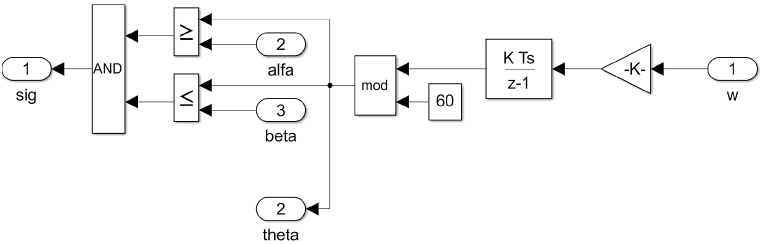
Speed estimator block Simulink model.

The speed is converted to Rotor position angle through position estimator block as shown in Fig. 15.

**Fig. 16:**
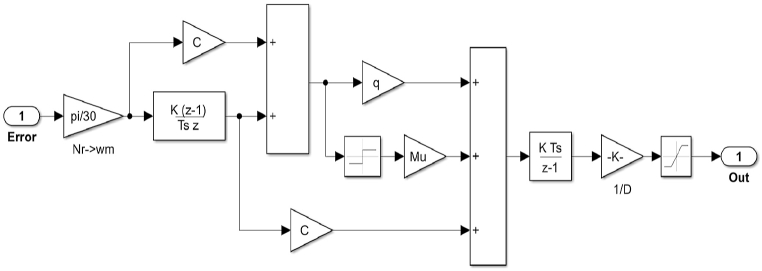
Sliding mode controller Simulink Model.

The sliding mode controller is developed for the switching function (s) and control function U(t) as shown in Fig. 16.

## 4 Proposed fuzzy logic controller

### 4.1 Fuzzy logic control

**Fig. 17:**
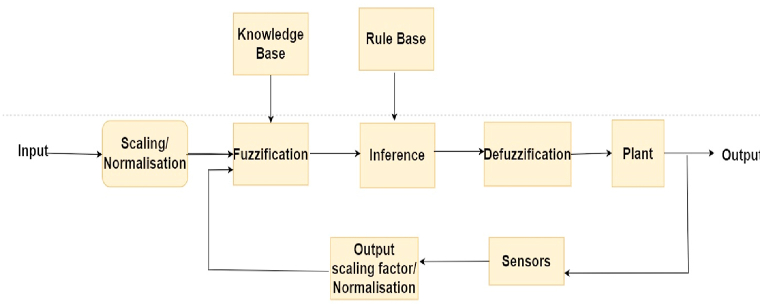
Block diagram of fuzzy controller.

Real-world situations can be extremely complex. The ability of fuzzy logic controllers to incorporate experience, logical reasoning, intuition, and heuristics into the system rather than relying on mathematical models is their primary advantage. Fig. 17 depicts a simplified block diagram of a traditional fuzzy controller. The characteristic function of the classical set assigns a value of 1 or 0 (Binary set) to each individual in the universal set, distinguishing between members and non-members of the crisp set under consideration. A fuzzy set is one that contains elements with varying degrees of membership in the set. In fuzzy set theory, various types of membership functions are commonly used for fuzzification of inputs. A set’s membership function describes it uniquely by mapping each element to a membership value between 0 and 1. The membership function is determined based on experience and problem specificity.

Prior to transforming crisp sets to fuzzy sets, the inputs are scaled and normalized. Fuzzification is the process of converting crisp values to fuzzy values. The conversion of fuzzy values is represented by membership functions. Membership function is designated as μ.

### 4.2 Design of MATLAB Simulink Fuzzy logic controller model

**Fig. 18:**
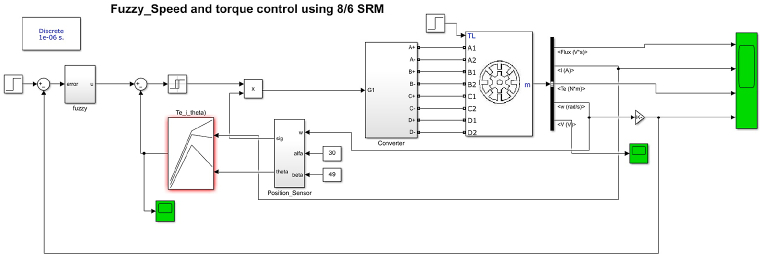
SRM control with fuzzy logic controller Simulink model.

It is created a MATLAB Simulink model for Fuzzy logic control of an 8/6 SRM drive as shown in Fig. 18. For closed loop control, the speed encoder feedback signal and current feedback are obtained.

**Fig. 19:**
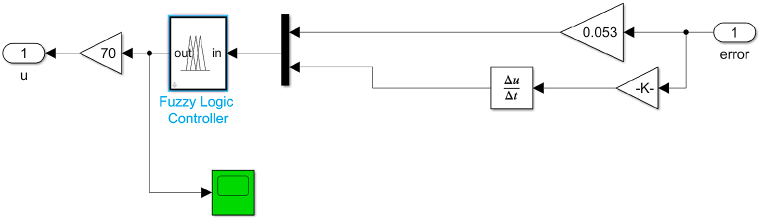
Fuzzy logic controller simulink model.

The Fuzzy controller is shown in Fig. 19. The speed error (ω – ωref) represented by E and Error change $\frac{d\left(\omega \;–\;\omega ref\right)}{dt}$ represented by EC are the crisp inputs to the fuzzy controller system and the output defuzzified crisp output is multiplied by a proportional gain to generate the control output.

**Fig. 20:**
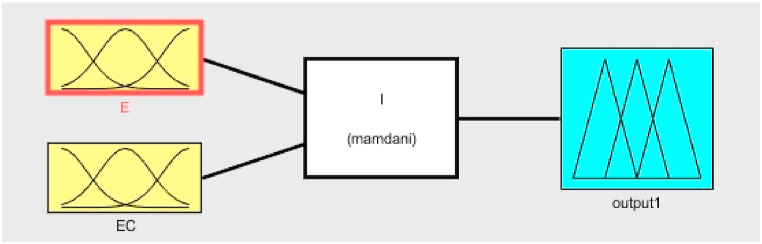
Fuzzy input-output membership functions.

The fuzzy inference engine is a process that uses if-then rules and fuzzy mechanisms to link input fuzzy sets to output fuzzy sets in order to get a reasonable result from fuzzy inputs. The Mamdani type and the Sugeno type are two forms of fuzzy inference systems. The Mamdani model shown in Fig. 20 is used in this simulation. Mamdani fuzzy systems use rule base for fuzzy input sets for determining the output distribution. The design of fuzzy controller system is as follows,

• Determining a set of fuzzy rules

The inputs and outputs are all divided into five fuzzy subsets: [NB,NM, NS, ZE, PS, PM,PB], where NB, NM, NS, ZE, PS, PM and PB mean negative big, negative medium, negative small, zero, positive small, positive medium and positive big, respectively.

• Fuzzifying the inputs using the input membership functions

The input and output membership functions are triangular with Z and S shapes at the ends. For operation (AND), the minimum value is used, and the centroid method is used for de-fuzzification. Combining the fuzzified inputs according to the fuzzy rules to establish rule strength. The rules consist of 49 IF-THEN rules. The E is the error and EC is the rate of the error change. The fuzzy rules are tabulated in Table 1.

**Table 1:** Fuzzy rule base for the proposed controller.

&		ERROR CHANGE (EC)						
		NB	NM	NS	ZE	PS	PM	PB
**ERROR (E)**	NB	NB	NB	NB	NB	Z	Z	PS
	NM	NB	NB	NB	NM	Z	Z	PM
	NS	NB	NB	NM	NS	Z	PS	PB
	ZE	NB	NM	NS	Z	PS	PM	PB
	PS	NM	NS	Z	PS	PM	PB	PB
	PM	NM	Z	Z	PM	PB	PB	PB
	PB	NS	Z	Z	PB	PB	PB	PB

**Fig. 21:**
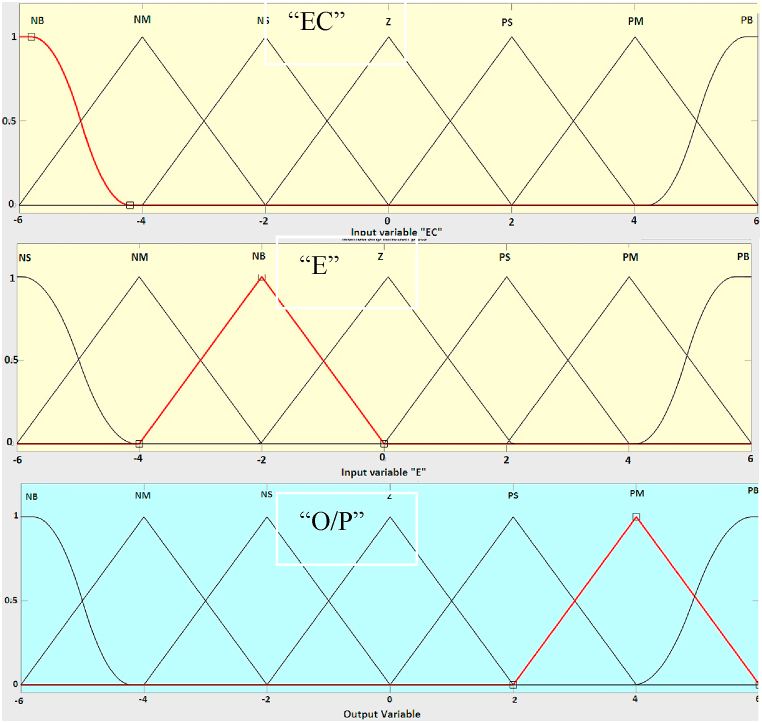
Inputs Error, Error Change and Output Fuzzy membership functions.

The input and output membership functions are shown in Fig. 21.

**Fig. 22:**
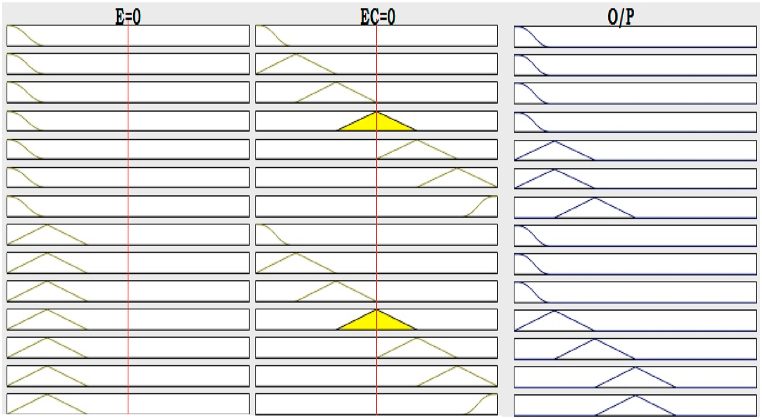
Graphical Representation of fuzzy rule base.

The input and output fuzzification rule base in graphical form is shown in Fig. 22.

**Fig. 23:**
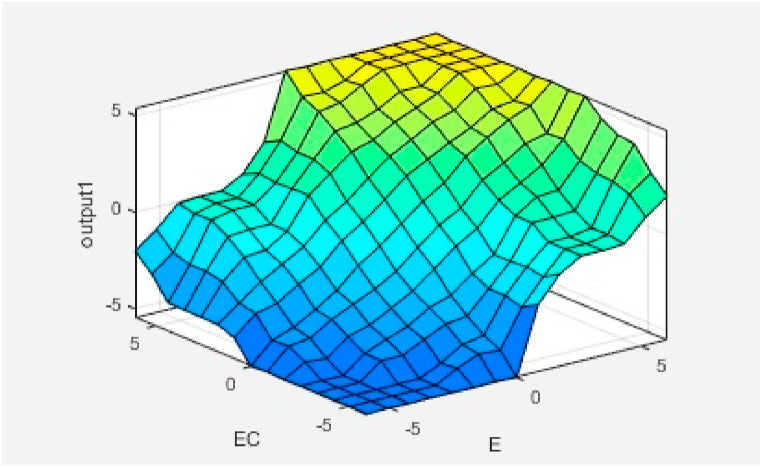
Fuzzy input-output surface viewer.

The rule strength and the output membership function are combined together to determine the rule’s consequence. The Surface Viewer of the input variables (E, EC) and the output variable (U) is shown in Fig. 23. The Rule consequences are combined to get an output distribution and the output is defuzzified using centroid method.

## 5 Simulation results

In this paper, the controller is developed for a 4 phase 8/6 poles rated 50 kW prototype SRM. The machine parameters are given in Table 2.

**Table 2:** Machine design parameters.

Parameter	Design data
Power	50 kW
Stator resistance	1.3 Ohm
Inertia	0.0013 kg m^2^
Friction	0.02 N m s
Unaligned inductance	1.167 mH
Aligned inductance	12.87 mH
Saturated Aligned Inductance	0.625 mH
Maximum current	120 A
Maximum flux linkage	0.32 Wb

### 5.1 SRM control with SMC controller

**Fig. 24:**
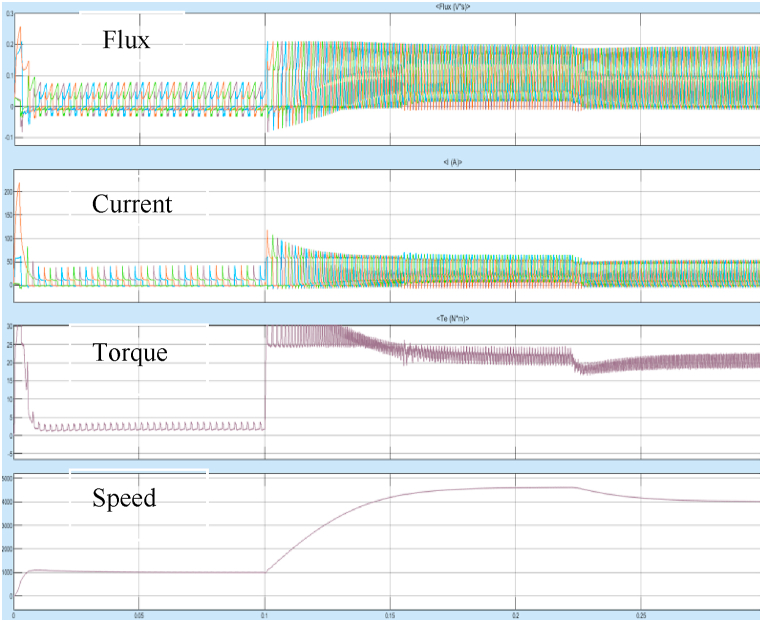
Starting response with SMC controller.

The starting response of the 8/6 SRM with sliding mode controller is shown in Fig. 24, the starting performance is better with SMC control. However for change in speed signal or disturbances, the controller overshoot and steady state error is more. The optimization of gain parameters was done and the better results were obtained.

**Fig. 25:**
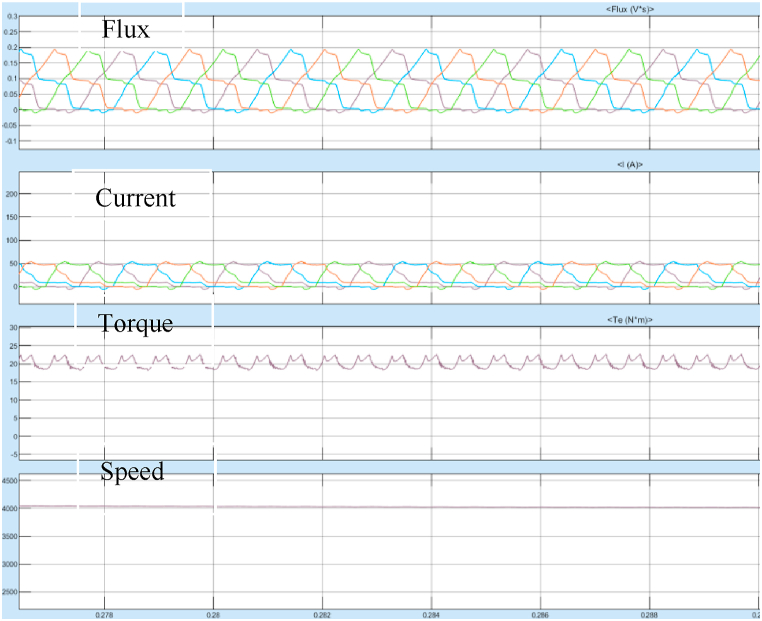
Steady state SRM output parameters at 4000 rpm with SMC controller.

The results of Steady State performance of SRM Drive at 4000 rpm is shown in Fig. 25.

The optimal switching angle was iteratively found out through simulation and it was observed, when the switching angle θon and θoff fixed at 30⁰ and 49⁰ respectively, the torque pulsations were minimum.

**Fig. 26:**
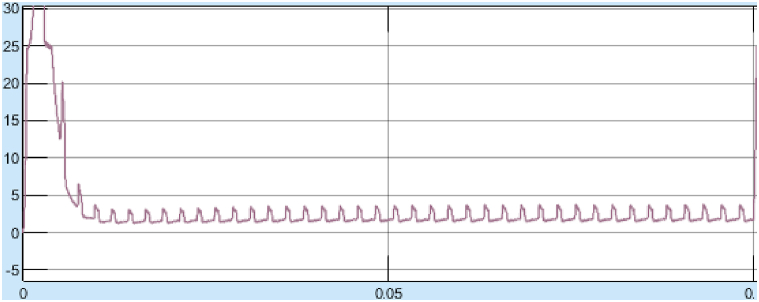
Steady state response of SRM torque at 1000 rpm with SMC controller.

**Fig. 27:**
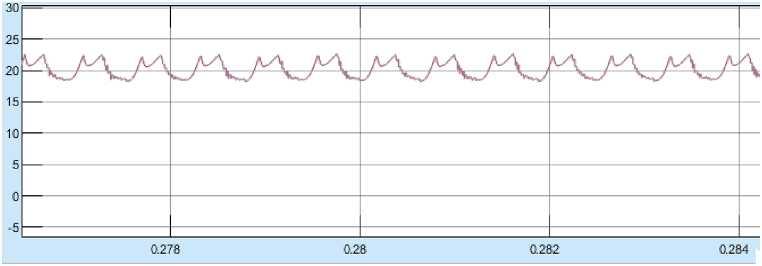
Steady state response of SRM torque at 4000 rpm with SMC controller.

The Torque oscillation is observed between 19 and 23 Nm at 4000 rpm. The Torque ripple is 19%. The starting and steady state response at 1000 rpm speed as shown in Fig. 26, Fig. 27.

### 5.2 With Fuzzy controller

**Fig. 28:**
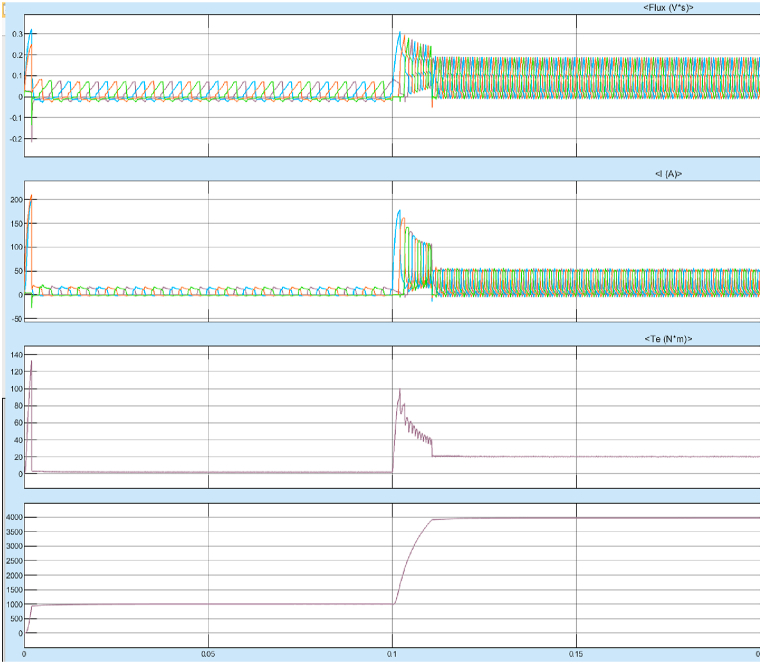
Starting response with fuzzy controller.

For the same machine parameters, the torque performance of 8/6 SRM with Fuzzy logic controller was simulated and the starting response of the 8/6 SRM with FLC is shown in Fig. 28.

**Fig. 29:**
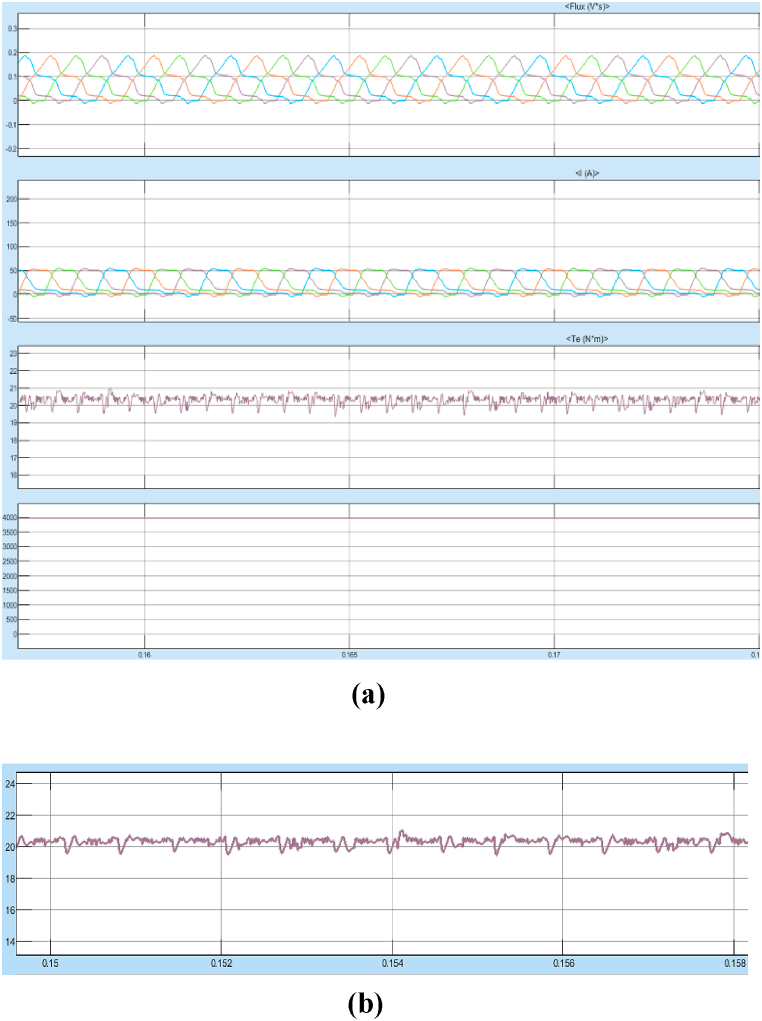
(a)Steady state response of SRM output parameters at 4000 rpm with fuzzy controller. (b)SRM torque at 4000rpm with fuzzy controller.

Fig. 28 shows the starting response of the motor is very smooth with reduced overshoots and lesser rise time. The steady state error after reaching the stable operating point is also very less. The system is more stable in case of any sudden change in input or any external disturbances. The results of Steady State performance of SRM Drive at 4000rpm with fuzzy controller is shown in Figs. 29a and 29b.

The Torque oscillation is observed between 19 and 19.5 Nm at 4000 rpm as observed in Fig. 29b.

## 6 Results and discussion

At lower speeds, the SMC controller produces torque of 3–4 Nm with high pulsation (28%), whereas in the case of fuzzy controller the torque pulsation is only 7%. The comparative results at 1000rpm and 4000rpm for SMC and Fuzzy controller are presented in Table 3.

**Table 3:** Comparison of results with SMC and fuzzy controller.

Speed (rpm)	SMC Controller		FUZZY Controller	
	Torque Oscillation (Nm)	Ripple (%)	Torque Oscillation (Nm)	Ripple (%)
1000	3-4 Nm	28%	3–3.2	7%
4000	19-24 Nm	19%	19.5–20.5	5%

The torque performance is superior with Fuzzy controller and also the torque ripple is very less compared to Sliding mode controller.

## 7 Conclusion

The paper compares the performance of an 8/6 SRM with two different controllers, a sliding mode controller and a fuzzy logic controller. The simulation results at mid and high speeds show that the fuzzy controller outperforms the SMC controller in terms of torque performance and torque ripple. We used a 2D lookup table with a known motor model in this study to calculate the estimated torque at various rotor positions and stator currents. For improved results, additional research will be conducted to implement an online torque estimator with a universal SRM mathematical model. The simulation results show that the proposed SRM drive with fuzzy controller effectively reduces steady state errors on any disturbance as well as torque ripple. The method performs well over a wide speed range.

### Author contribution statement

S. Kudiyarasan: Conceived and designed the experiments; Performed the experiments; Analyzed and interpreted the data; Contributed reagents, materials, analysis tools or data; Wrote the paper.

N. Sthalasayanam & Vijayalakshmi Karunakaran: Conceived and designed the experiments; Performed the experiments; Analyzed and interpreted the data; Contributed reagents, materials, analysis tools or data.

## Funding statement

This research did not receive any specific grant from funding agencies in the public, commercial, or not-for-profit sectors.

## Data availability statement

Data will be made available on request.

## Declaration of interest’s statement

The authors declare no conflict of interest.
